# Electroconvulsive Therapy for Persistent Postural Perceptual Dizziness: A Case Report

**DOI:** 10.1155/crps/9996688

**Published:** 2025-04-10

**Authors:** Natalie Seiler, Michelle McAlary, Richard Kanaan, Revi Nair

**Affiliations:** ^1^Department of Psychiatry, The University of Melbourne, Melbourne, Australia; ^2^Division of Mental Health, Austin Health, Melbourne, Australia

**Keywords:** ECT, electroconvulsive therapy, functional disorders, persistent postural perceptual dizziness, PPPD, vestibular disorders

## Abstract

Persistent postural perceptual dizziness (PPPD) is a functional vestibular disorder that can cause significant distress and impairment. Evidence for effective PPPD treatment is still limited and among suggested treatment regimes there have been mixed findings regarding neurostimulation. We present a case report of electroconvulsive therapy (ECT) for an individual with severe PPPD. The patient was a middle-aged woman admitted for acute suicidal ideation in the setting of peripartum depression and PPPD, following several unsuccessful antidepressant trials and a prior suicide attempt. She provided voluntary consent for ECT during admission, and 10 acute bifrontal ECT treatments were completed. The patient was substantially improved in PPPD, depressive, and anxiety symptoms, as well as quality of life. ECT may be beneficial in addressing PPPD symptoms. Further research is needed regarding the role of neurostimulation in PPPD.

## 1. Introduction

Persistent postural perceptual dizziness (PPPD) is a chronic functional vestibular disorder [1], which is often considered as a form of functional neurological disorder [2]. The Bárány Society guidelines require that five criteria are met for diagnosis—(a) dizziness, unsteadiness, or non-spinning vertigo present for most days, for at least 3 months (b) symptoms can occur at rest, but are exacerbated by standing, walking, movement, or complex visual stimuli exposure (c) precipitants may include disorders that induce unsteadiness, vertigo, balance, or dizziness issues, including vestibular diseases, neurological or other medical conditions, or psychological distress (d) symptoms cause significant distress or functional impairment, and (e) symptoms cannot be better explained by another condition [1]. PPPD can significantly impact quality of life [3], and disability can vary from minimal to severe impairment [1]. Individuals have reported experiencing loss of identity and confidence in terms of functioning, difficulties in terms of sense-making, dismissal from healthcare providers, poor psychological well-being and sense of helplessness, and dissociative experiences related to disorientation and dizziness [4]. PPPD is a clinical diagnosis, and there are no pathognomonic signs on physical examination, pathology, or imaging. It has only recently been defined; however, symptoms have been reported in the literature for many years [1].

PPPD may be precipitated by vestibular or medical causes or psychological conditions including panic attacks or severe anxiety [1]. Following the precipitant, there may be a central adaptation process that contributes to long-term symptoms of dizziness or imbalance due to interpretation changes of visual and somatosensory input [5]. The original insult may lead to the overuse of visual cues and maladaptive postural control techniques. When there is impaired vestibular functioning, postural control relies more on visual and somatosensory inputs. After resolution of the precipitant this compensatory mechanism should cease, however in PPPD, individuals may experience hypervigilance and continue to overly rely on visual and somatosensory inputs to maintain postural stability. This results in maladaptive responses to mobilization and visual cues, and contributes to dizziness and other symptoms [6]. Comorbidities may include vestibular disorders or psychiatric conditions such as anxiety, posttraumatic stress disorder [6], or depression [7].

Evidence for PPPD treatment efficacy is limited and is an area of ongoing investigation [5]. Currently, a multimodal approach is recommended, including cognitive behavioural therapy (CBT), vestibular rehabilitation physiotherapy, and serotonergic medications such as selective serotonin reuptake inhibitors (SSRIs) or selective serotonin and norepinephrine reuptake inhibitors (SNRIs) [7].

Electroconvulsive therapy (ECT) is the application of electrical charges through scalp electrodes to the brain to elicit a generalized epileptic seizure. ECT has evidence as treatment for psychiatric conditions including psychotic disorders, catatonia, mania, suicidal ideation, and depression [8]. ECT is often delivered under brief general anesthesia, muscle relaxants, and continuous medical monitoring. Most initial treatment courses are 6–12 treatments over 2–4 weeks. Common side effects include self-limiting aches, myalgias, nausea, vomiting, fatigue, and cognitive impairment. Most patients will have mild to moderate cognitive side effects, which often resolves days to weeks after the last treatment [9]. While ECT can be life-saving, it may be underused in some settings due to lack of knowledge among the public and healthcare providers, stigma, and media sensationalism [9]. To the best of our knowledge, there has been no previous research in relation to the use of ECT in PPPD. We present a case study of an individual with PPPD who experienced improvements in PPPD symptoms following ECT treatment.

## 2. Case Presentation

Jane*⁣*^*∗*^ (Pseudonym used) was a woman aged in her forties who was admitted to Austin Health with severe peripartum depression and PPPD.

Jane lived at home with her husband and children. Prior to the onset of PPPD, she was high functioning and was engaged in part-time employment alongside managing the household. After the development of PPPD symptoms, Jane required extended periods of sick leave and struggled with domestic and community activities of daily living.

Jane's PPPD symptoms had been present for 11 months at time of admission and had gradually worsened. Jane reported significant psychosocial stressors and a premorbid anxious personality at onset but denied organic triggers. She had previously been reviewed by an ear, nose, and throat surgeon, a neurophysiotherapist, and several neurologists, and workup had included physical examinations, vestibular testing, and neuroimaging. A diagnosis of PPPD had been confirmed by these teams and she had trialed cyroheptadine, pizotifen, doxylamine, and prochlorperazine to no benefit. She had also trialed sertraline, escitalopram, mirtazapine, agomelatine, and desvenlafaxine for the treatment of PPPD and depressive symptoms, but these were ceased due to side effects or inefficacy. Jane saw a physiotherapist and a chiropractor on several occasions without improvement. With the exception of PPPD and the investigations needed to confirm the diagnosis, there was no significant medical history.

Two to 3 months after the onset of PPPD, Jane became pregnant and developed depressive symptoms which she attributed to the PPPD and fears regarding how she would cope as a mother while experiencing constant dizziness and related symptoms. This culminated in self-injury and a subsequent inpatient psychiatric admission. Jane denied any prior psychiatric history or mental health complications in previous pregnancies. There was also no diagnosed mental illness in the family.

### 2.1. Medications

On admission, Jane was on venlafaxine 150 mg in the morning for the treatment of depression and PPPD, olanzapine 5 mg twice daily for anxiety and sleep, propranolol 5 mg twice daily for anxiety symptoms, melatonin 7 mg nighttime for sleep, temazepam 10 mg nighttime as needed for sleep, and nutritional and herbal supplements. She denied any therapeutic benefits from this regime. Propranolol was ceased but medications were otherwise continued during admission and ECT.

### 2.2. Symptomatology

On admission, depressive symptoms included low mood, reduced pleasure in activities, poor sleep and energy levels, concentration difficulties, and ruminations in relation to guilt, worthlessness, hopelessness, and helplessness. Jane experienced intense and constant suicidal ideation which she attributed to her PPPD symptoms. She endorsed significant anxiety in relation to PPPD, with apprehension and fear regarding how she would cope and recover.

On admission, PPPD symptoms included constant daily dizziness, subjective unsteadiness, and non-spinning vertigo, worse from sitting to standing but also present at rest. On mobilization she felt as though her head and body were not synchronized with her environment, “like being on a rollercoaster”. The dizziness made it difficult to think clearly and she often felt disoriented and detached from her body and environment. She experienced infrequent light-headedness, and denied nausea or vomiting, aura, headaches, hearing or vision changes, or neurological symptoms. There was no history of falls or mobility difficulties. Her symptoms caused severe constant distress, made her reluctant to leave her home, and she felt pessimistic regarding her ability to function in the future.

During hospitalization, Jane was observed at times to be perplexed and blunted with significant fixation on PPPD symptoms, and psychotic depression remained a primary differential. Jane denied any psychotic, manic, or organic symptoms, and no other psychiatric or physical symptoms were elicited upon systems review.

### 2.3. Mental State Examination

Jane was a well-groomed and casually dressed woman who appeared of stated age, with reasonable engagement throughout assessment. Speech was spontaneous with normal mechanics. She was dysthymic, with a mildly perplexed affect with restricted range and reactivity. Her mood was low, her thought form was linear and goal-directed but persevering in relation to PPPD symptoms and subsequent suicidal ideation. Nil perceptual abnormalities were elicited. She was oriented with regard to time, person, and place, with an intact insight and judgment, and aware of mental health needs and help-seeking in relation to treatment.

### 2.4. Physical Examination

Jane's cardiorespiratory, abdominal, neurological, and cranial nerve examinations were normal upon admission. Vital signs including lying and standing blood pressure and heart rate remained largely within normal limits throughout admission.

### 2.5. Investigations

Investigations showed a mild iron deficiency which was treated with iron supplementation. Non-contrast magnetic resonance imaging (MRI)-Brain and C Spine and MRI venogram showed non-specific, minor periventricular, right frontal, and left superior frontal gyral white matter T2/fluid-attenuated inversion recovery (FLAIR) hyperintensities but no other abnormalities.

### 2.6. Inpatient Progress

During admission, 10 bifrontal ECT treatments occurred over 3.5 weeks in the setting of acute suicidality and severe depression. Electroencephalogram (EEG) results largely showed optimal seizure duration, progression, interhemispheric concordance, spike-wave amplitude/spike-wave/slow wave regularity, and post-ictal suppression. Side effects included mild, self-resolving headaches and cognitive impairment including short-term memory loss.

There was a marked improvement in PPPD symptoms after six ECT treatments, with reduced intensity and frequency of dizziness, subjective unsteadiness, and non-spinning vertigo, and resolution of detachment symptoms. Additionally, PPPD symptoms were reported as less salient, and Jane was more able to focus on daily activities. There were several times per a day on most days where she did not notice the dizziness. These improvements contributed to greater confidence, hopefulness, and quality of life, and Jane reported that her depressive symptoms and apprehensive anxiety resolved in the setting of this.

Outcome measures were taken before and after the course of 10 ECT treatments (Table 1). Dizziness Handicap Inventory (DHI) scores [10] improved from 28 to 6 and European Evaluation of Vertigo (EEV) scale scores [11] improved from 12 to 11 in terms of motion intolerance. Vertigo Symptom Scale (VSS) (short form) scores [12] were unchanged at 12, where positive responses included item 4 (sensation of spinning lasting >20 min daily), item 6 (sensation of dizziness or disorientation all day) as Jane reported some disorientation from ECT and occasional days with constant dizziness, and item 10 (unsteadiness for >20 min daily). Although Jane reported that her sensation of spinning and unsteadiness had improved from constantly present to only present intermittently during the day, this was not captured in the VSS scales. Health of the Nation Outcome scale (HoNOS) score [13] improved from 26 to 11 and Global Assessment of Functioning (GAF) scale score [14] improved from 30 to 80 (Table 1).

Subjective improvements were observed after seven treatments and continued to improve up to 10 treatments. Objective improvements on mental state examination were observed after four treatments, with Jane appearing brighter, euthymic, more motivated, and spontaneous in engagement. At discharge, there were marked improvements in depressive symptoms including mood, motivation, concentration, sleep, and energy levels, and resolution of negative ruminations, anhedonia, and suicidal ideation. Montgomery Asberg Depression Rating Scale (MADRS) score [15] improved from 44 to 4 and General Anxiety Disorder 7 item scale (GAD-7) score [16] improved from 16 to 3 over 3.5 weeks (Table 1).

In terms of medication management, near discharge venlafaxine was increased to 225 mg daily to mitigate relapse risk in relation to PPPD and peripartum depression following cessation of ECT. Olanzapine was also increased to 15 mg nighttime to support with sleep and due to the differential diagnosis of psychotic depression. Propranolol and temazepam were ceased during admission, and sumatriptan, betahistine, and compression stockings were trialled and ceased due to inefficacy. Throughout admission, close family liaison and multidisciplinary management were undertaken. The discharge plan included referrals to vestibular physiotherapy and CBT for ongoing PPPD treatment.

## 3. Discussion

In this case study, PPPD symptoms including frequency and intensity of dizziness, non-spinning vertigo, and subjective unsteadiness, alongside distress, functioning, and depressive and anxiety symptoms, all markedly improved in response to ECT. MADRS, GAD-7, DHI, EEDV, and HoNOS and GAF scores improved following ECT treatment. The VSS scores remained unchanged as Jane's primary symptoms and sensations of motion, spinning, and unsteadiness, when they did occur, could still last more than 20 min per an episode. However, they occurred less frequently, were less salient, and no longer occurred daily.

To the best of our knowledge, this is the first reported application of ECT to improve PPPD symptoms, which occurred in the setting of depression treatment. There has been previous reports of functional disorder symptoms improving with ECT, including among refractory presentations with comorbid mood disorders [17]. Other forms of neurostimulation in PPPD have been reported, including repetitive transcranial magnetic stimulation (rTMS), which may improve dizziness, anxiety, and depressive symptoms when used in conjunction with SSRI/SNRI medication and vestibular physiotherapy rehabilitation training [18], whereas a study using transcranial direct current stimulation (tDCS) showed no significant improvements [19]. Noninvasive vagus nerve stimulation has shown benefits in terms of dizziness severity, quality of life, depressive and anxiety symptoms, and body sway measures during postural challenges [20]. Ongoing research in this area would further elucidate the interactions between depression, anxiety, and PPPD. Psychiatric comorbidities among people who experience PPPD have included higher rates of depression, anxiety, posttraumatic stress disorder, and obsessive compulsive personality traits [6]. In this case, the PPPD symptoms preceded depressive and anxiety symptoms, and PPPD was the primary condition that resulted in a secondary depression. Further research is needed to explore whether ECT is a modality, which may directly treat PPPD, or if optimizing psychiatric comorbidities such as depression through treatments such as ECT may improve PPPD symptoms.

The mechanism of action of ECT for psychiatric disorders is still not fully understood. ECT may increase gray-matter volume in frontolimbic areas and the integrity of white matter tracts within the frontal and temporal lobes. ECT may also increase monoamine neurotransmitter release, produce neurogenesis, normalize cortisol release [9], normalize glutamate/glutamine levels, increase synaptic connectivity, and normalize cerebral blood flow to the frontal lobes [21]. The understanding of brain changes in PPPD is also still developing. Structural changes in gray matter may include the visual cortex, multisensory vestibular cortices, cerebellum, and areas of the brain implicated with anxiety, whereas there are mixed findings in relation to white matter changes in PPPD. Abnormal functional activation and connectivity within the vestibular cortex, including parieto-insular vestibular cortex, cerebellum, visual cortex, and areas of the brain implicated in anxiety, have also been reported [22]. In the setting of current limited evidence for the best treatment approach [5], research regarding whether ECT may be able to normalize some areas of brain dysfunction in PPPD is a key area of further development.

## 4. Conclusion

This case report describes an individual with depression and PPPD whose symptoms including dizziness, subjective unsteadiness, and non-spinning vertigo, alongside associated distress and functional impairment, all markedly improved in response to acute bifrontal ECT initiated for the treatment of depression. The role of neurostimulation in PPPD and associated psychiatric comorbidities would benefit from further investigation, particularly among individuals with treatment non-response or severe presentations.

## Figures and Tables

**Table 1:** Outcome measures before and after ECT treatment.

Scale	Before ECT	After ECT
Dizziness Handicap Inventory	28	6
European Evaluation of Vertigo Scale	12	11
Vertigo Symptom Scale (short form)	12	12
Montgomery Asberg Depression Rating Scale	44	4
General Anxiety Disorder (7 item)	16	3
Health of the Nation Outcome	26	11
Global Assessment of Functioning	30	80

## Data Availability

Deidentified data that support the findings of this study are available on request from the corresponding author, N.S. The complete data are not publicly available due to research participant privacy.
